# Mutational study of sapovirus expression in insect cells

**DOI:** 10.1186/1743-422X-2-13

**Published:** 2005-02-23

**Authors:** Grant S Hansman, Kazuhiko Katayama, Tomoichiro Oka, Katsuro Natori, Naokazu Takeda

**Affiliations:** 1 Department of Virology II, National Institute of Infectious Diseases, Tokyo, Japan

## Abstract

Human sapovirus (SaV), an agent of human gastroenteritis, cannot be grown in cell culture, but expression of the recombinant capsid protein (rVP1) in a baculovirus expression system results in the formation of virus-like particles (VLPs). In this study we compared the time-course expression of two different SaV rVP1 constructs. One construct had the native sequence (Wt construct), whereas the other had two nucleotide point mutations in which one mutation caused an amino acid substitution and one was silent (MEG-1076 construct). While both constructs formed VLPs morphologically similar to native SaV, Northern blot analysis indicated that the MEG-1076 rVP1 mRNA had increased steady-state levels. Furthermore, Western blot analysis and an antigen enzyme-linked immunosorbent assay showed that the MEG-1076 construct had increased expression levels of rVP1 and yields of VLPs. Interestingly, the position of the mutated residue was strictly conserved residue among other human SaV strains, suggesting an important role for rVP1 expression.

## Introduction

The family *Caliciviridae *is made up of four genera, *Sapovirus*, *Norovirus*, *Lagovirus*, and *Vesivirus*, which contain sapovirus (SaV), norovirus (NoV), rabbit hemorrhagic disease virus, and feline calicivirus strains, respectively. Human SaV and NoV strains are agents of gastroenteritis. The prototype strain of human SaV, the Sapporo virus, was originally discovered from an outbreak of gastroenteritis in an orphanage in Sapporo, Japan, in 1977 [1]. Chiba et al. identified viruses with the typical animal calicivirus morphology, called the "Star of David" structure, by electron microscopy (EM). SaV strains were recently divided into five genogroups (GI to GV), of which GI, GII, GIV, and GV strains infect humans, while GIII strains infect porcine species [2]. The SaV GI, GIV, and GV genomes are each predicted to contain three main open reading frames (ORFs), whereas SaV GII and GIII have two ORFs. SaV ORF1 encodes for non-structural proteins and the major capsid protein (VP1). SaV ORF2 (VP2) and ORF3 (VP3) encoded proteins of yet unknown functions. The NoV genome is organized in a slightly different way than the SaV, since ORF1 encodes all the nonstructural proteins, ORF2 encodes the capsid protein (VP1), and ORF3 encodes a small protein (VP2).

Human SaV and NoV strains are noncultivable, but expression of the recombinant VP1 (rVP1) in a baculovirus expression system results in the self-assembly of virus-like particles (VLPs) that are morphologically similar to native SaV [3,4] In a recent NoV expression study, a single amino acid substitution in the rVP1 gene affected VLP formation but not rVP1 expression [5]. In a different study, inclusions of NoV ORF3 and poly(A) sequences in a construct increased the expression levels of NoV rVP1 and the stability of VLPs when compared to constructs without these sequences [6]. Recently, cryo-EM analysis of SaV VLPs and X-ray crystallography analysis of NoV VLPs predicted the SaV shell (S) and protruding domains (subdomains P1 and P2) that were based the NoV domains [7,8]. Chen et al. also described strictly and moderately conserved amino acid residues in the capsid protein among the four genera in family *Caliciviridae*.

The purpose of this study was to compare the time-course expression of two different SaV rVP1 constructs in a baculovirus expression system by Northern blotting, Western blotting, enzyme-linked immunosorbent assay (ELISA), and EM. Our novel results have indicated that nucleotide point mutations increased the yields of SaV VLPs in insect cells, offering an alternative explanation for the increased expression levels of rVP1 and yield of VLPs.

## Results

### Wt, MQG-1076, and MEG-1076 constructs

**Figure 1:**
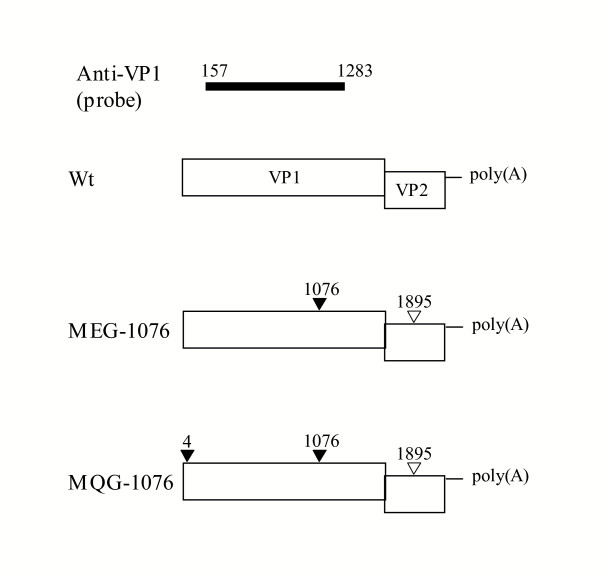
Schematics of the SaV constructs, Wt, MEG-1076, and MQG-1076, containing the rVP1, rVP2, and poly(A) sequences. Each construct began at the predicted AUG start. The triangles show the positions of the nucleotide point mutations. The black triangle had an amino acid substitution in the VP1, whereas the open triangle in the VP2 gene did not change amino acid sequence. An RNA probe (anti-VP1) was used to monitor the transcription of rVP1 mRNA in which contained the native sequence, i.e., lacking the mutation at 1076.

Expression of SaV rVP1 in a baculovirus expression system results in the self-assembly of VLPs [4]. However, during PCR amplification nucleotide point mutations occurred in our initial MQG-1076 construct, at nucleotide positions 4 and 1076 in VP1, which resulted in two amino acid substitutions at residues 2 and 358, respectively, and a silent nucleotide mutation at position 1895 in VP2 (Fig. 1). Despite these two substitutions the MQG-1076 construct formed VLPs morphological similar to native SaV (data not shown). In order to further investigate these substitutions we expressed another construct (MEG-1076 construct) having only one substitution, at residue 358 in VP1 (Fig. 1). This construct also formed VLPs. Finally we expressed a construct (Wt construct) without these nucleotide point mutations, i.e., having the native sequence. The Wt construct also formed VLPs, however the expression level of rVP1 was noticeably lower than those of the MQG-1076 and MEG-1076 constructs in which had similar levels (data not shown). In order to compare expression levels, we infected Wt and MEG-1076 recombinant baculoviruses each at a multiplicity of infection (MOI) of 14.5 in 2.7 × 10^6 ^confluent Tn5 cells in 1.5 ml of Ex-Cell 405 medium followed by incubation at 26°C. RNA transcription and rVP1 expression experiments were run in parallel for the Wt and MEG-1076 constructs.

### Northern blot analysis

**Figure 2:**
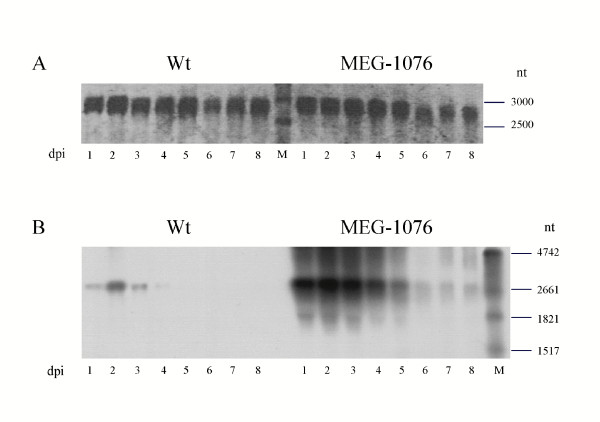
Northern Blot analysis of Wt and MEG-1076 rVP1 mRNA. The total RNA was purified from the cells at 1, 2, 3, 4, 5, 6, 7, and 8 dpi. (A) The relative amounts of total RNA for each construct. (B) The steady-state levels of rVP1 mRNA with an anti-VP1 probe specific for the VP1 gene, corresponding to the VP1 nucleotide position 157 to 1283.

Total RNA was extracted from the cells at 1, 2, 3, 4, 5, 6, 7, and 8 days postinfection (dpi) for Wt and MEG-1076 constructs. Equal amounts (500 ng) of total RNA were added to a 2% agarose gel containing formaldehyde and stained with SYBR Gold (Fig. 2A). The rVP1 mRNA was then analysed by Northern blot with a probe specific for the VP1 gene (native sequence) corresponding to the VP1 position 157 to 1283 (Fig. 1). The rVP1 mRNA transcript was predicted to be approximately 2300 nucleotides long. As shown in Figure 2B, rVP1 mRNA was detected for each construct. This result showed that the insert sequence and some part of the baculovirus vector, approximately 300 nt, was transcribed, although the exact location(s) on the vector has yet to be determined. Nevertheless, the MEG-1076 construct had increased band intensities, indicating an increased steady-state level, when compared to those of the Wt construct (Fig. 2B). For the Wt construct, rVP1 mRNA was detected at 1 dpi, peaked at 2 dpi, decreased at 3 and 4 dpi, and then decreased to undetectable levels at 5, 6, 7, and 8 dpi. For the MEG-1076 construct, rVP1 mRNA was detected at 1 dpi, peaked at 2 dpi, had steady-state levels at 3 and 4 dpi, and then decreased at 5 dpi but could still be detected at 6, 7, and 8 dpi. These results indicated that the MEG-1076 rVP1 mRNA also had greater stability when compared to those of the Wt rVP1 mRNA.

### Western Blot analysis

**Figure 3:**
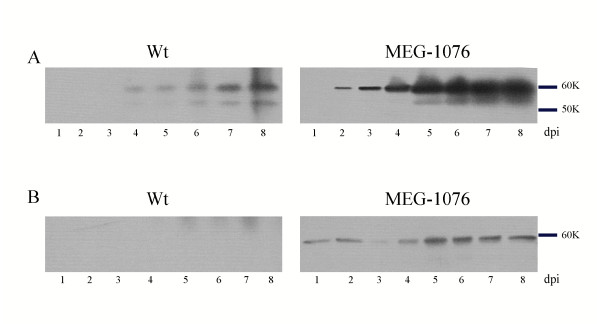
Western blot analysis of Wt and MEG-1076 rVP1. Confluent Tn5 cells were infected with Mc114 recombinant baculoviruses at MOI of 14.5 and incubated at 26°C. The culture medium, including the cells, were harvested 1, 2, 3, 4, 5, 6, 7, and 8 dpi as described in the materials and methods. (A) The cell culture medium was concentrated by ultracentrifugation, resuspended in 20 μl of Grace's medium, and 5 μl was mixed with loading dye and loaded into each well. (B) The cell lysate was separated from the culture medium, resuspended in 200 μl of Grace's medium, and 5 μl was mixed with loading dye and loaded into each well.

Western blot analysis was used to compare the expression levels of Wt and MEG-1076 rVP1. The culture medium was separated from the cell lysate 1, 2, 3, 4, 5, 6, 7, and 8 dpi as described in the Materials and Methods. Equal volumes of culture medium and cell lysate at each dpi were used for both constructs. Proteins were separated by SDS-PAGE, electrotransferred to PVDF, and detected with a 1:3000 dilution of hyperimmune rabbit Mc114 VLP antiserum. A band at the predicted rVP1 size (60 K) was first detected in the culture medium at 2 and 4 dpi for MEG-1076 and Wt constructs, respectively, which increased each day thereafter as evidenced by an increase in band intensity (Fig. 3A). As indicated by increased band intensities, the MEG-1076 construct expressed increased levels of rVP1 (60 K) than those of the Wt construct. Similarly, these results were reproduced using different MOIs in order to address the variability in virus stock quality (data not shown).

A thin band of approximately 55 K was also detected in the culture medium that appeared at 4 and 5 dpi for Wt and MEG-1076 constructs, respectively, and increased each day thereafter. In a different experiment, we determined the amino acid sequence of the MQG-1076 upper and lower bands by an Edman's degradation method. We discovered that the first three amino acid residues were MQG for both the upper and lower bands. This result indicated that the 55 K bands for these constructs were likely truncated or C-terminal deleted forms of rVP1. A thin band of 60 K was detected at every dpi in the cell lysate for the MEG-1076 construct (Fig. 3B), however the intensity of this band did not increase to the same extent as the MEG-1076 60 K band in the culture medium (Fig. 3A). This suggested that immediately after translation the majority of rVP1 was rapidly exported from the cells to the culture medium, though a fraction accumulated within the cells. This may also explain why no 60 K bands were detected in the cell lysate for Wt construct.

The VP2 amino acid sequence was the same in all constructs. We did not detect rVP2 during the time-course expression of the MQG-1076 construct using the antiserum raised against *E. coli *expressed VP2 (data not shown).

### Antigen ELISA and EM analysis of Wt and MEG-1076 VLPs

**Figure 4:**
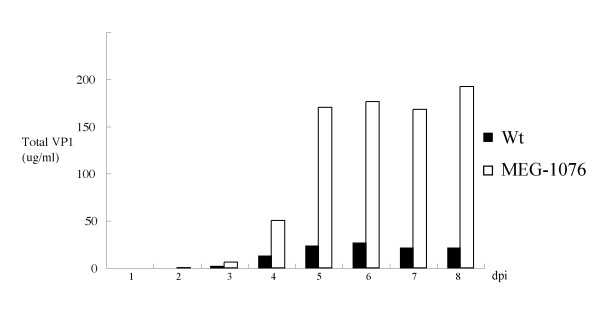
Antigen ELISA analysis of Wt and MEG-1076 VLPs. The ELISA used hyperimmune rabbit (capture) and guinea pig (detector) antiserum raised against Mc114 VLPs. For the antigen ELISA, purified Mc114 VLPs were used as the positive control at concentrations ranging from 500 ng to 0.24 ng.

An antigen ELISA system was used to compare the yields of Wt and MEG-1076 VLPs at 1, 2, 3, 4, 5, 6, 7, and 8 dpi. The ELISA incorporated hyperimmune rabbit (capture) and guinea pig (detector) antisera raised against purified Mc114 VLPs [4]. The ELISA first detected VLPs at 2 and 3 dpi for MEG-1076 and Wt constructs, respectively (Fig. 4). For both constructs, the yields of VLPs increased each day thereafter, however the MEG-1076 construct had increased yields of VLPs than those of the Wt construct at 4, 5, 6, 7, and 8 dpi, approximately 6-fold increase. EM was used to verify the VLP formation of each of these constructs. We first detected VLPs at 4 dpi in the culture medium for both constructs and the numbers of VLPs increased each day thereafter (data not shown).

### Amino acid analysis

**Figure 5:**
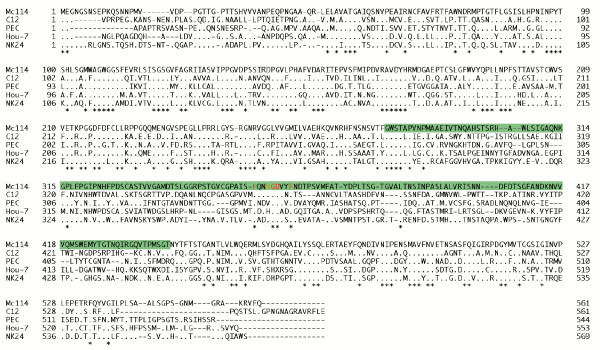
VP1 amino acid alignment of SaV GI, GII, GIII, GV, and GV strains. We originally aligned 21 SaV GI, GII, and GV sequences but to simplify the figure we used one representative strain from each genogroup. The green bar shows the SaV P2 domain predicted by Chen et al. [7]. The asterisks indicate conserved amino acids. We originally aligned 21 different VP1 amino acid sequences of SaV GI, GII, and GV strains and found the residue (N) at position 358 (yellow) was strictly conserved (data not shown), but SaV GIII and GIV strains (PEC and Hou-7, respectively) had other residues at this position. The alignment of the five SaV genogroups showed the amino acid motif, GD, was strictly conserved (red) and several other amino acids surrounding the residue at position 358 were also strictly conserved (red).

The MEG-1076 construct contained a nucleotide point mutation in which resulted in an amino acid substitution at position 358 in VP1. We aligned 21 different VP1 amino acid sequences of SaV GI, GII, and GV strains and found this residue was strictly conserved, but more importantly, there was a strictly conserved amino acid motif at this site, NGDV (data not shown). However, when we included a porcine SaV GIII strain and a recently identified SaV GIV strain (PEC and Hou-7, respectively), only the GD site was strictly conserved, though several other amino acids nearby were also strictly conserved (Fig. 5). Further analysis of other SaV GIV strains are clearly needed in order to examine the possibility that the NGDV motif was moderately conserved in other human SaV strains. Figure 5 also showed that the predicted SaV P2 domain had very few conserved amino acid residues. Apart from the strictly conserved GD motif, the only other strictly conserved motif in the P2 domain was at the 5' end.

## Discussion

Expression of the human SaV rVP1 in a baculovirus expression system was first reported in 1997 [9]. In that study, the full-length VP1 gene, ORF2, and poly(A) sequences were included in a construct (Sapporo strain, GI). The second human SaV reported to form VLPs was with a construct (Houston/90 strain, GI) using only the VP1 sequence, i.e., lacking ORF2 and poly(A) sequences [10], while the third human SaV reported to form VLPs used a construct (Parkville strain, GI) with only VP1 and ORF2 sequences, i.e., lacking poly(A) sequence [7]. We recently expressed human SaV GI, GII, and GV rVP1 with constructs (Mc14, C12, and NK24 strains, respectively) that included ORF2 and poly(A) sequences [4]. Additional information on human SaV rVP1 expression is lacking, although it appeared that the yields of human SaV VLPs were typically low for these three genogroups.

In this study, we compared the time-course expression of two different Mc114 SaV rVP1 constructs in a baculovirus expression system (Fig. 1). The MEG-1076 construct had two nucleotide point mutations, one in the VP1 gene in which resulted in an amino acid substitution, and one in the VP2 gene in which was silent. Although both constructs formed VLPs morphological similar to native SaV, the levels of transcription, translation, and VLP formation were clearly different. As shown in Figure 2B, the MEG-1076 rVP1 mRNA had increased steady-state levels and greater stability when compared to those of the Wt rVP1 mRNA. This difference was understood to be due to the nucleotide mutations in the MEG-1076 construct, since a similar result was observed in a NoV expression study [6]. Bertolotti-Ciarlet et al. found that a nucleotide point mutation in a NoV rVP1 construct (ORF2-*A****U****G → A****C****G*-ORF3+3' UTR construct, represented in bold) had decreased levels of rVP1 mRNA at 36 hours post-infection, by approximately 50%, when compared to a construct without the mutation (ORF2+ORF3+3' UTR construct). Bertolotti-Ciarlet suggested that the RNA secondary structure or changes in the mRNA stability could be responsible for the different steady-state levels, but this was not proven.

Also, the MEG-1076 construct had increased levels of rVP1 expression and yields of VLPs in the culture medium when compared to those of the Wt construct (Fig. 3A). On the other hand, the concentration of rVP1 in the cell lysate remained more or less the same during the time-course expression for the MEG-1076 construct. And for the Wt construct, rVP1 was not detected in the cell lysate, although this may have been related to the low expression levels (Fig. 3B). Our results showed that the MEG-1076 construct had a 6-fold increase in yields of VLPs in the culture medium (Fig. 4), which corresponded to approximately 80 μg of CsCl purified VLPs from 200 ml of culture medium (at 6 dpi), but less than 5 μg of CsCl purified VLPs in the cell lysate (data not shown). These results suggested that either (i) immediately after translation the majority of rVP1 was exported from the cells to the culture medium where the majority of VLPs were folded but a fraction were simultaneously folded within the cells or (ii) VLPs were folded within the cells and then the majority of VLPs were immediately exported from the cells to the culture medium, though a fraction remained within the cells.

In a recent NoV expression study, a single amino acid substitution in the rVP1 gene affected VLP formation but not rVP1 expression [5]. In that study, a (native) histidine residue at position 91 (relative to NoV Snow Mountain Virus strain amino acid VP1 sequence) was found to be essential for VLP formation and a construct with a substituted (mutant) arginine residue at this position failed to form VLPs despite expressing rVP1. Interestingly, that study found a single amino substitution was critical for the formation of VLPs, whereas our results showed that a single amino acid substitution was beneficial, i.e., increased the yields of VLPs. Bertolotti-Ciarlet found that inclusions of NoV ORF3 and poly(A) sequences in a construct increased the expression levels of NoV rVP1 and the stability of VLPs when compared to constructs without these sequences; and suggested that expression of other caliciviruses (NoV and SaV) rVP1 that resulted in low yields or unstable VLPs may be due to constructs that lacked the VP2 gene [6]. An alternative explanation was that point mutations influenced steady-state levels of mRNA and stability, which in turn influenced VLP formation. In our case, one or two nucleotide point mutations caused an enhancement of transcription, leading to increased yields of SaV VLPs in insect cells. Furthermore, many of these studies that expressed calicivirus rVP1 in insect cells only examined rVP1 expression and yields of VLPs but not rVP1 mRNA transcription [11-14]. However, another reason for the increased yields of VLPs may be associated with adaptation of SaV rVP1 to the baculovirus expression system and insect cells, since a similar result was observed with porcine enteric calicivirus in primary kidney cells [15].

Although the growth rate and replication efficiency of the recombinant baculoviruses themselves and differences in the levels of virus replication might account for such variation, we observed similar results using other MOIs, that is, the MEG-1076 construct continued to express greater yields of VLPs than the Wt construct (data not shown). Another explanation may have been differences in the extents to which these baculoviruses induce apoptosis and all these may result from features in the baculovirus skeleton rather than from the inserted SaV sequence. Such effects might for instance affect the number of adherent cells harvested or the degradation rates of both proteins and RNAs. However, we found that the MQG-1076 construct, developed from a separate experiment, had similar expression levels to that of the MEG-1076 construct (data not shown), which may eliminate the possibility that the baculovirus skeleton played a role in the increased yields of VLPs. On the other hand, we could not demonstrate whether the nucleotide mutations in VP1 and/or in ORF2 affected the transcription, a construct with only one of these mutations would be needed. Nevertheless, our results indicate that translation was exclusively affected by the single amino acid substitution in VP1. Therefore, the final increase in yields of VLPs may have been coupled at multiple levels, involving one or both of the nucleotide mutations in VP1 and VP2.

We did not detect rVP2 during the time-course expression of the MQG-1076 construct (data not shown). The Wt and MEG-1076 constructs had an identical amino acid sequence, which would suggest a similar negative-result. NoV studies have found that inclusion of VP2 increases the stability of VLPs, though the expression level of NoV rVP2 was low [6]. These results may suggest that (i) SaV rVP2 was expressed at undetectable levels, (ii) SaV rVP2 was not expressed in the insect cells, or (iii) SaV rVP2 was degraded in the insect cells. The SaV GI, GIV, and GV genomes are each predicted to encode a third ORF (ORF3) overlapping the VP1 gene, whereas SaV GII and GIII have only two ORFs. The functions of SaV ORF2 and ORF3 still remain unknown.

The amino acid substitution (N → S) for the MEG-1076 construct occurred in the VP1 gene at residue 358. This asparagine residue was recently identified as a moderately conserved residue among the caliciviruses capsid proteins [7], but more importantly, the residue was strictly conserved among 21 different SaV GI, GII, and GV strains and belonged to a strictly conserved amino acid motif, NGDV (Fig. 5). However, when we included SaV GIII and GIV strains (PEC and Hou-7, respectively) we found that only the GD amino acids were strictly conserved though several other amino acids nearby were also strictly conserved (Fig. 5). These data further suggested that this site played an important role in the regulation of SaV VLP formation.

Recently, the cryo-EM analysis of SaV was determined and compared to NoV X-ray crystallography structure [7]. Chen et al. analysed 30 different VP1 amino acid sequences of calicivirus strains belonging to the four genera in the family *Caliciviridae *and identified strictly and moderately conserved residues, and predicted the P1 and P2 domains of SaV VP1 based on NoV X-ray crystallography structure. Based on these predictions, the residue at position 358 (amino acid sequence) was found as a moderately conserved residue among the caliciviruses. This arginine residue was predicated to be in the P2 domain, which is defined as the outer most protruding domain for NoV and thought to provide strain diversity [16]. Further high-resolution structural analysis of SaV VLPs is clearly needed in order to determine the precise domains and regions of SaV. However, our expression results have indicated that only approximately 80 μg of purified VLPs from 200 ml of culture medium was possible (data not shown), thus in order to determine the X-ray crystallography structure of SaV, a minimum increase in expression level of about 20-fold would be required: a challenging feat.

## Materials and methods

### Virus strain, RNA extraction, cDNA synthesis

SaV GI Mc114 strain (GenBank accession number, AY237422) was isolated from a male infant seven months of age from the McCormic Hospital, Chiang Mai, Thailand on the 7th May 2001 [17]. RNA extraction and cDNA synthesis were performed as previously described [18].

### PCR and sequencing

Our initial SaV rVP1 construct (MQG-1076 construct) was amplified with ExTaq DNA polymerase. However, this construct was later found to have two nucleotide point mutations in ORF1 at positions 4 (**G**AG → **C**AG) and 1076 (A**A**T → A**G**T) and one nucleotide point mutation in ORF2 at position 1895 (GT**G **→ GT**A**) (relative to the VP1 start and represented in bold). Primer and PCR errors likely introduced these mutations. These three nucleotide point mutations resulted in two amino acid substitutions in the VP1 gene, one at the second residue, where glutamic acid (E) → glutamine (Q), and one at residue 358, where asparagine (N) → serine (S). The nucleotide point mutation in ORF2 did not result in an amino substitution. Despite the two amino acid substitutions, the MQG-1076 construct formed VLPs. We designed another construct (MEG-1076) using the pDEST8-MQG-1076 as template but with a new sense primer and used KOD-plus DNA polymerase according to the manufacture's instructions (Toyobo, Japan). The MEG-1076 construct had the same nucleotide point mutations at positions 1076 in VP1 and 1895 in VP2 as the MQG-1076 construct but not at nucleotide 4 in VP1 (Fig. 1). Lastly, we designed a third construct with the native sequence (Wt construct) using KOD-plus DNA polymerase and the original cDNA [4]. PCR-amplified fragments were cloned into the Gateway Expression System (Invitrogen, Carlsbad, Calif.) as previously described [4]. The insert sequences of the pDONR8 plasmids were confirmed, including the partial upstream and downstream sequences on the plasmids in which were found to be identical for the Wt and MEG-1076 constructs. Sequencing was performed as previously described [18].

### Expression of rVP1 in insect cells

Recombinant bacmids were transfected into Sf9 cells (Riken Cell Bank, Japan) and the recombinant baculoviruses was collected as previously described [4]. The expression of the rVP1 constructs were analyzed by infecting recombinant baculoviruses at a MOI of 14.5 in 2.7 × 10^6 ^confluent Tn5 cells in 1.5 ml of Ex-Cell 405 medium followed by incubation at 26°C. The total culture medium was harvested 1, 2, 3, 4, 5, 6, 7, and 8 dpi. The culture medium was centrifuged for 10 min at 3,000 × *g*, and further centrifuged for 30 min at 10,000 × *g*. The VLPs in the culture medium were further concentrated by ultracentrifugation for 2 h at 45,000 rpm at 4°C (Beckman TLA-55 rotor), and then resuspended in 30 μl of Grace's medium. The cell lysate from the first centrifuge was resuspended in 200 μl of Grace's medium and stored at 4°C.

### Northern blotting

Total RNA was prepared from the attached cells at 1, 2, 3, 4, 5, and 6 dpi with 1 ml of Isogen (Nippon Gene, Japan). For 7 and 8 dpi, the cell culture medium (containing unattached cells) was collected and centrifuged for 5 min at 3,000 × *g*, the supernatant removed, and then the cells were dissolved with 1 ml of Isogen. The cells were stored at -80°C. RNA was purified by a chloroform/ ethanol method (Nippon Gene, Japan). Briefly, RNA was mixed with chloroform, centrifuged at 12,000 × *g *for 15 min at 4°C, and the aqueous layer collected. This was repeated once, and then the aqueous layer collected and mixed with isopropanol and stored overnight at -20°C. The solution was mixed, centrifuged at 12,000 × *g *for 15 min at 4°C, and the supernatant discarded. The pellet was resuspended in 80% ethanol, centrifuged at 12,000 × *g *for 15 min at 4°C. This was repeated once, and then the pellet air-dried and resuspended in 25 μl of TE, and stored at -80°C. The amounts of purified RNA were determined spectrophotometrically (Bio-Rad, USA). The same amounts (500 ng) of total RNA were loaded for each construct and each dpi onto a 2% denaturing agarose gel containing formaldehyde. The amounts of total RNA were compared using SYBR Gold staining (Invitrogen, USA). RNA was transferred to a positively charged nylon transfer membrane (Hybond-N+; Amersham Biosciences, Ireland) under vacuum (VacuGene XL; Pharamacia LKB, Sweden) and analyzed by Northern blotting according to the DIG Northern Starter Kit (Roche, USA), except for a minor modification. Briefly, a RNA probe corresponding to Mc114 VP1 position 157 to 1283 (anti-VP1) was generated from a PCR fragment (native sequence) according to the manufacture's instructions (Roche, USA). Hybridization was performed overnight at 68°C with anti-VP1 in 10 ml of ultrasensitive hybridization buffer (Ambion, Canada). After hybridization, immunological detection was performed according to the manufacture's instructions (Roche, USA).

### Western blotting, ELISA, EM, and protein sequencing

Western blotting, ELISA, and EM were used to examine rVP1 expression as previously described [4]. However, it should be acknowledged that the hyperimmune rabbit and guinea pig antisera were raised against the MQG-1076 VLPs. Protein sequences were determined by an Edman's degradation method.

### Amino acid alignment

VP1 nucleotide sequences were translated using Genetyx software (software development Co. Version 11.2.2) and submitted to online ClustalW at DDBJ . In total, we aligned different 21 SaV GI, GII, GIII, GIV, and GV sequences, and included: Arg39, AY289803; Bristol, AJ249939; C12, AY603425; Cruise ship/00, AY289804; PEC, AF182760; Dresden, AY694184; Hou-7, AF435814; Houston/86/US, U95643; Houston/27/90/US, U95644; London/29845/92/UK, U95645; Lyon/598/97/F, AJ271056; Manchester, X86560; Mc2, AY237419; Mc10, AY237420; Mex340/1990, AF435812; Mex14917/00, AF435813; NK24, AY646856; Parkville, U73124; Potsdam, AAG01042; Plymouth, X86559; Sapporo/82/Japan, U65427; and Sakaeo-15, AY646855.

## Competing interests

The author(s) declare that they have no competing interests.

## Authors' contributions

GH carried out the study and wrote the manuscript. KK, TO, KN, and NT participated in the design of the study and helped to draft the manuscript.
